# Charring-induced morphological changes of Chinese “Five Grains”: An experimental study

**DOI:** 10.3389/fpls.2023.1063617

**Published:** 2023-02-03

**Authors:** Yang Liu, Yang Xi, Fei Zhang, Zhenzhen Wang, Can Wang, Shiyong Yu, Xuexiang Chen

**Affiliations:** ^1^ Joint International Research Laboratory for Environmental and Social Archaeology, Institute of Cultural Heritage, Shandong University, Qingdao, China; ^2^ Department of Cultural Heritage Registration, the Administrative Office of Humble Administrator’s Garden, Suzhou, China; ^3^ Shandong University Museum, Shandong University, Qingdao, China; ^4^ Department of Archaeology, School of History and Culture, Shandong University, Jinan, China; ^5^ School of Geography, Geomatics, and Planning, Jiangsu Normal University, Xuzhou, China

**Keywords:** Chinese “Five Grains”, charring, morphological change, experimental study, archaeobotany

## Abstract

**Introduction:**

Charring process affects the preservation potential of seeds, resulting in limited perceptions of crop assemblages recovered from archaeological layers. Therefore, the specifics of the charring process deserve further investigation. Colloquially referred to as the “Five Grains” (五谷), bread wheat (*Triticum aestivum*), foxtail millet (*Setaria italica*), broomcorn millet (*Panicum miliaceum*), rice (*Oryza sativa*), and soybean (*Glycine max*) represent a set of four major cultivated cereals and a pulse constituting crucial staple food in Chinese history and the most frequently discovered crops at archaeological sites in China

**Methods:**

This paper aims to understand the changes in size, volume, and weight loss of grains under variable aerobic charring conditions. The size and weight were measured for the untreated specimens and the specimens heated at different temperatures and over different time-periods.

**Results:**

We found that temperature and exposure time directly affected the grain size. Specifically, the grains of most species shrank at lower temperatures and expanded rapidly at higher temperatures.

**Discussion:**

Among the “Five Grains”, soybean was the type least affected by charring, followed by wheat, rice, and millet. Volume and weight can be used as conversion factors to minimize the bias in quantitative representation due to varied charring preservation potential. For rice, wheat and soybean, the variation in volume is smaller. For millet, both volume and weight can be used as the control to understand the consequences of charring for the assemblage. Further experiments and comparisons of ancient samples are needed in future studies to investigate other factors that affect the preservation of charred plant remains.

## 1 Introduction

Grain cultivation has always been the top priority for agrarian societies (Bellwood, 2005; Crawford, 2017). In ancient China, the country was referred to as “sheji” (社稷), the collective name for the Earth God and the Harvest God, which shows the significance of land and grain to Chinese civilization. In ancient Chinese literature, important crops are usually grouped and colloquially called “Five Grains”, “Six Grains” or “Nine Grains”, though there are still many debates about the specific types of grains they represent (Min, 1982; Song, 2002). Among these groupings, the term “Five Grains” (五谷), common in modern Chinese, generally refers to a set of five cultivated crops, including bread wheat (*Triticum aestivum*), foxtail millet (*Setaria italica*), broomcorn millet (*Panicum miliaceum*), rice (*Oryza sativa*), and soybean (*Glycine max*), which represent the four essential cereals and a pulse constituting staple food in Chinese history; they account for the largest proportion of plant remains in archaeological sites (Zhao, 2005). The idea of “Five Grains” first appeared in documents from the Eastern Zhou Dynasty (2996 – 2721 BP) (Song, 2002). According to archaeological findings, the “Five Grains” appeared as early as the Longshan period (4300 – 3800 BP) (Zhao, 2020). The “Five Grains” still represent the major crops that account for more than 90% of food production in China. Polyculture farming can increase the productivity of agriculture, improve land-use efficiency, and increase disaster resilience (Zhao, 2011). Therefore, the multi-crop cultivation may correspond to the formation of Chinese civilization, which is an important issue in archaeology.

Plant remains usually cannot be preserved at an archaeological site for long periods of time because of their unstable chemical and physical conditions and microbiological degradation. However, their macrofossils (like charred, desiccated, or waterlogged finds) and/or microfossils (like phytoliths and starch granules) can be well preserved in archaeological contexts, among which charred seeds are the most commonly found type. Charring is an incomplete combustion process that can make seeds resistant to biological degradation and microbial attack, while causing their morphological change (Styring et al., 2013; Charles et al., 2015; Dong et al., 2022). Charred seeds have long been used in archaeobotany to infer ancient arable economy and plant consumption. However, evidence has shown that preservation conditions and the charring process have different effects on different grains.

There is often disagreement between the interpretation of charred plant remains and other proxies such as phytoliths and crop stable isotopes in terms of human diet (Wang et al., 2015a; Lu, 2017). For example, during the Neolithic, millets represented the primary staple in northern China. The studies of when and where they were domesticated, and how they spread are important topics in local and regional archaeology (Zhao, 2005; Hunt et al., 2008; Lu et al., 2009; Deng et al., 2018; Martin et al., 2021). Based on the analysis of charred grains, scholars concluded that foxtail millet outnumbered broomcorn millet in the Central Plain by the Late Peiligang/Early Yangshao period (8000 – 6000 BP) (Lee et al., 2007). On the other hand, the phytolith results showed that broomcorn millet was still the dominant crop during the Longshan period at some sites in the Central Plain (Zhang et al., 2010; Wang et al., 2018). Therefore, whether the quantity of charred seeds can be used to infer the productivity of arable economy or the composition of human diet is an open question. Quantitative analysis of macrobotanical remains needs to be cautiously approached (Boardman and Jones, 1990). A number of solutions have been proposed, such as correcting the absolute counts using the weight or volume, which can reflect the grain yield. One of the methods uses the number of each unearthed crop and the average weight/volume of 1000 modern grains from each crop, which can help convert the absolute number of each crop to allow a comparison, and thus determine which crop dominates (2021; Zhang et al., 2010; Zhou et al., 2016; Sheng et al., 2018; Li et al., 2022b). By establishing formulas that estimate the original grain weight of wheat and barley using the length*width and length*thickness of charred seeds, Ferrrio offered another method for estimating crop output while reducing the impact of charring (2006; Ferrio et al., 2004). By comparing the converted proportion of weight or volume, it is possible to further understand the importance of different crops, and reconstruct ancestral crop selection.

Charring process is one of the crucial factors determining the type, amount and plant part of crop species preserved in archaeological contexts. It is, therefore, pertinent to ask the following question: is there any bias caused by different seed preservation potential with respect to charring? The critical aspects are the condition and process of charring, because seeds may be completely or partially charred under the influence of different factors. In the context of daily human activities, sheaves of harvested crops could be unexpectedly heated before threshing, dropped during food preparation and cooking, buried in the ground and heated by fire above 200°C on the floor or preserved through other taphonomic processes (Schiffer, 1972; van der Veen, 2007; Aldeias et al., 2016).

Simulation experiments have been carried out to understand the charring process of seeds of crops, weeds, and fruits. Most of these studies focus on the morphological changes, while the others demonstrate chemical alternations such as fractionations in carbon and nitrogen stable isotopes (Boardman and Jones, 1990; Gustafsson, 2000; Wright, 2003; Braadbaart et al., 2004; Guarino and Sciarrillo, 2004; Braadbaart and van Bergen, 2005; Braadbaart et al., 2005; Braadbaart, 2007; Braadbaart et al., 2007; Märkle and Rösch, 2008; Fraser et al., 2013; Charles et al., 2015; Nitsch et al., 2015; Aldeias et al., 2016; Walsh, 2017; Castillo, 2019; Su et al., 2019; White et al., 2019; Biswas et al., 2020; Wang and Lu, 2020; ). Temperature and time are the most significant factors, while factors such as oxidizing/anoxic conditions, moisture content of the plant part, heating rate, distance from the fire source, burial depth, and the un/shelled status can also influence the charring (White et al., 2019; Li et al., 2022a). Moreover, they tend to have different charring effects on different species. For example, Chinese and Indian millets exhibit significant differences in shrinkage after charring (Castillo, 2019).

Experimental evidence has shown that the seed preservation and identifiable features retention of millet grains under oxidizing conditions are better than in reducing conditions (Wright, 2003; Märkle and Rösch, 2008; Walsh, 2017), while other experiments have found that some species (such as wheat) are better preserved under reducing conditions (Ferrio et al., 2004; Sievers and Wadley, 2008). In some experiments, the temperature was strictly controlled for various species. However, the same or comparable conditions have not been tested for the traditional Chinese “Five Grains”. Therefore, we conducted a simulation experiment aimed at monitoring the charring process of “Five Grains” heated at different temperatures and over different periods. Our charring experiment was conducted under aerobic conditions.

Our goal was to explore various temperature ranges and the corresponding changes in size, weight and shape of the grains during the charring process. In addition, statistical analysis was conducted to quantify the significance of these changes in the resulting grains under varying conditions. The L/W ratio, weight, and volume were analyzed to determine whether the charring had the same effect on these different parameters. By “reversing” the charring process, with the “Five Grains” as an example in a laboratory setting, we attempt to provide the knowledge of the factors affecting the preservation of charred seeds. Such knowledge can contribute to the reconstructions of ancient human diets, arable agriculture, and ecology.

## 2 Materials and methods

### 2.1 Materials

The primary materials in this work include mature grains of rice, broomcorn millet, foxtail millet, soybean, and bread wheat. The wheat samples were taken from a farmland in Wushe County, Jiaozuo, Henan Province, and the grains and husks were separated by hand. The other samples were purchased from a local supermarket (Jinan, Shandong Province, China), already dehusked and with their origin declared as Yangming District, Mudanjiang City, Heilongjiang Province. All of these regions are in northern China, which have a temperate continental climate with little intra-regional variation in planting conditions. Therefore, we selected samples from these regions for comparison.

### 2.2 Charring experiments

We conducted three tests based on previous studies (2007; Braadbaart, 2004; Braadbaart et al., 2004; Märkle and Rösch, 2008; Wang and Lu, 2020). It was found that the charring temperature of roughly between 240-300°C and the charring period of 1-5 hours were suitable for all five crops. Therefore, we used these temperature and time ranges in the formal experiments.

The weight of grains was measured and compared before and after charring at different temperatures and times. First, the grains were weighed and then placed into crucibles, which were kept at 30°C for a month to minimize the effects of grain moisture content variability. Then, these crucibles were heated stepwise in a staged-program muffle furnace (CV-VULCAN 3-550) to 240°C, 250°C, 260°C, 270°C, 280°C, 290°C, and 300°C. The grains heated to the same temperature were divided into three groups, which were kept at the end temperature for 1 h, 3 h, and 5 h, respectively. Thus, for each species, the grains were divided into 21 groups, and each group contained 20 grains. The initial constant heating rate was 10°C/min, rising until the desired temperature was reached.

### 2.3 Sectioning and microscopic observation

**Figure 1:**
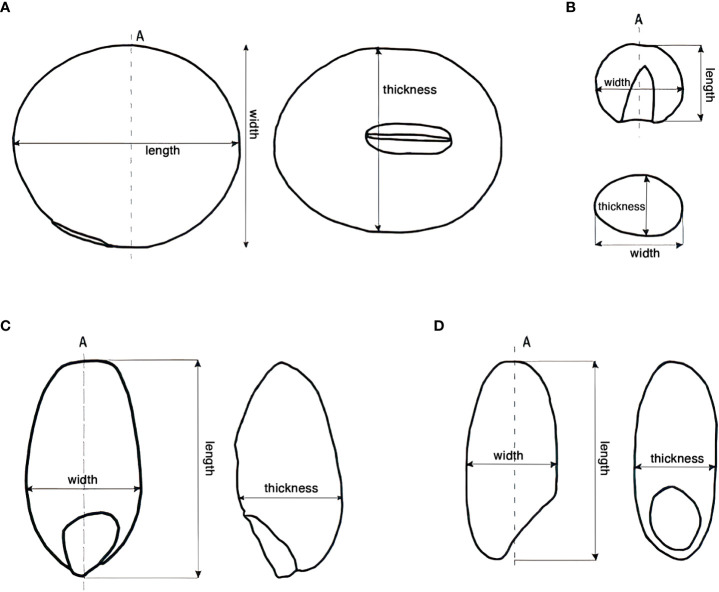
Cutting direction and measurement of the grains. **(A)** Soybean; **(B)** Millet; **(C)** Wheat; **(D)** Rice.

First, the unheated and the charred grains were observed under a Nikon C-FMC stereomicroscope. The morphological changes of the grains at different temperatures and heating durations were documented with the Nikon digital camera system. Second, samples from the simulation experiment and the Beitaishang archaeological site were cut longitudinally along the direction of the dotted line A (indicated in **Figure 1**); we first made a shallow cut along this line, and then force-split the grain, in order to get a ‘natural’ surface of the fracture and thus be able to observe the starch structure under the SEM. The seeds were adhered to the conductive resin and plated with gold. Then, the essential parts such as episperm, pericarp, embryo, and endosperm were observed with a scanning electron microscope (JEOL JCM-6000).

### 2.4 Measuring morphological traits

The geometry of all samples, including weight, length, width, and thickness, was measured to a precision of 0.001 gram or millimeter before and after heating. The measurement method is shown in **Figure 1**. Since we wanted to conduct a volume analysis later, we estimated the volume using the formula specified below, even though the irregular shape did not allow us to calculate the exact volume. The volume here only represents an estimated value, in order to combine the length, width, and thickness for the calculation.

The volume of the grains except soybean is calculated and presented as length×width×thickness. The shape of soybean is close to an ellipsoid, and thus the volume was calculated using the following formula: 4/3π×half of the length×half of the width×half of the thickness. The density of the grains is also calculated and presented as weight/volume.

### 2.5 Statistical analysis

To explore whether different charring temperatures and times caused statistically significant changes in the grain morphology, MANOVA (Multivariate ANOVA) analysis was conducted. The parameters analyzed include: length variation, width variation, thickness variation, L/W ratio variation, and volume variation of the “Five Grains” before and after charring. The analysis was conducted using the SPSS computer program (ver. 26).

## 3 Results

The average measurement results of the unheated and heated grains of “Five Grains” are shown in **Table S1**. For later discussion, charring is defined as the stage in which the color of the grains turns brown or black, the grain is accompanied by slight or clear distortion with identifiable traits, the dissolution of starch granules intensifies, and the pores between starch granules gradually increase (Charles et al., 2015; Dong et al., 2022). The original dataset is shown in **Table S2**. The detailed statistical results and charring condition of each group are shown in **Table S3**. The morphology changes after heating are summarized as follows.

### 3.1 Alteration of morphology

**Figure 2:**
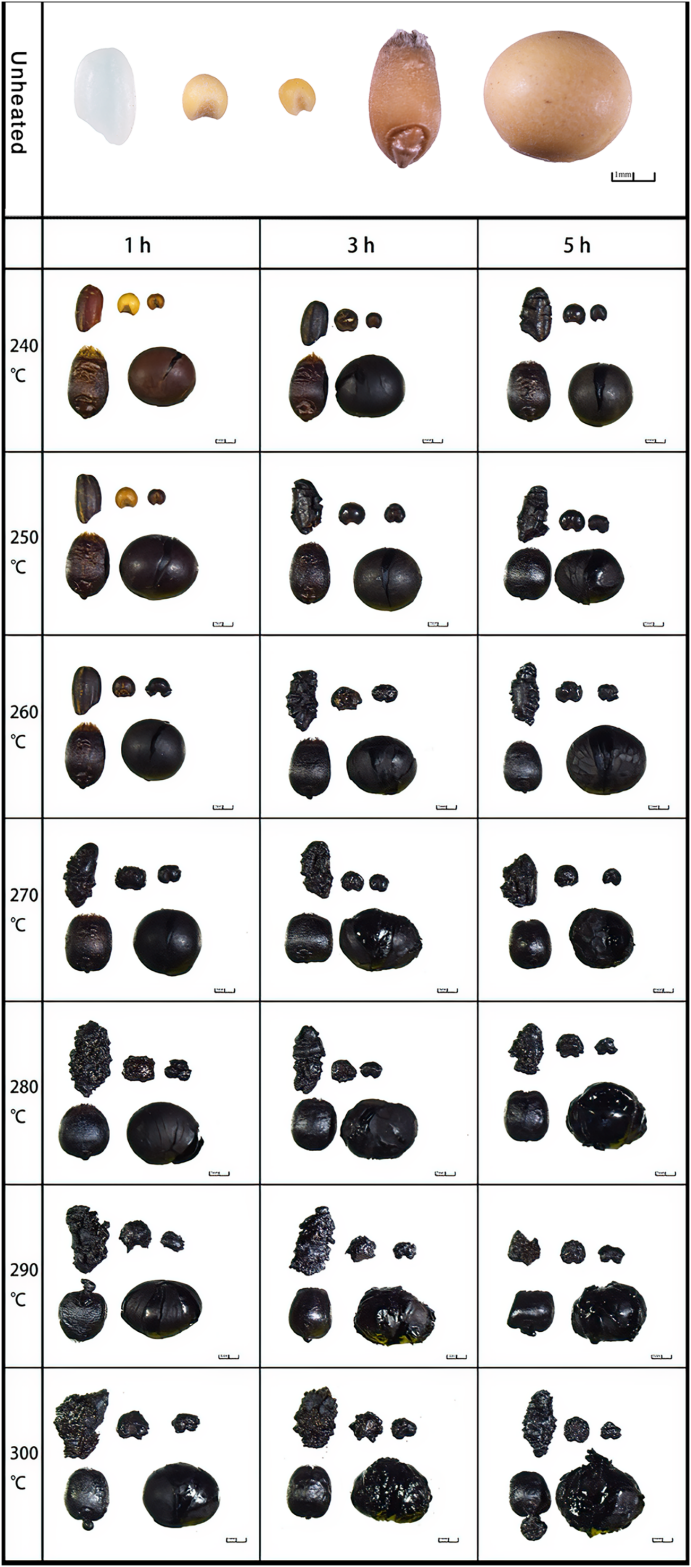
Morphology of the “Five Grains” at different temperatures and duration of heating. (In each picture from left to right: above: rice, broomcorn millet, foxtail millet; below: wheat, soybean).

The morphological changes of the “Five Grains” after the heating process are shown in **Figure 2**. In terms of color, the seeds changed from yellow to brown and then to gray and black. In brief, the grains became darker and shinier with the increase of time and temperature, and the damage became more severe, with more noticeable deviations from the initial form.

**Figure 3:**
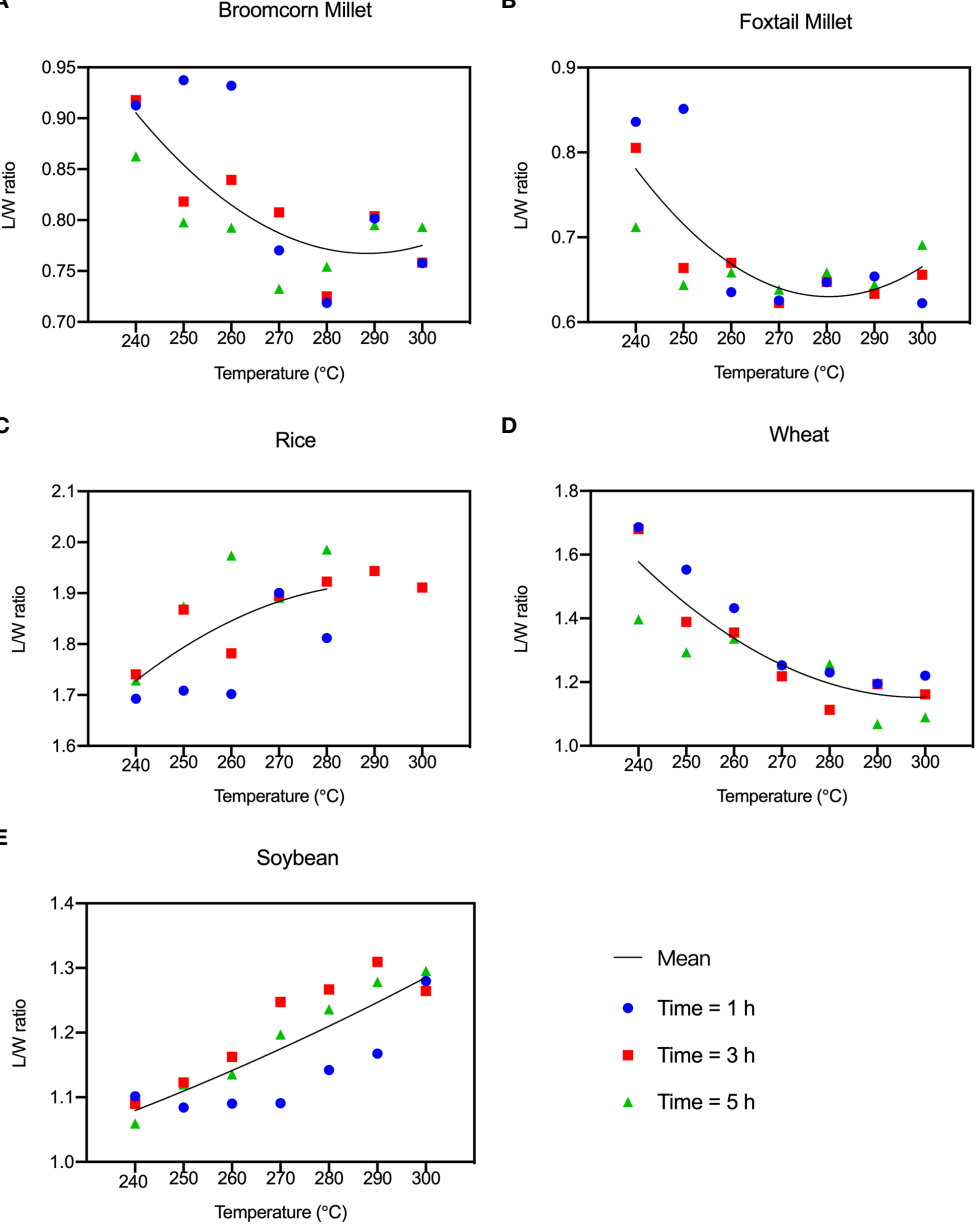
L/W ratio of the “Five Grains” at different temperatures and times during the heating process. **(A)** Broomcorn millet; **(B)** Foxtail millet; **(C)** Rice; **(D)** Wheat; **(E)** Soybean.

The length and width of the grains changed with the increase of temperature and time (**Figure 3**). Before heating, wheat had the highest length to width ratio (L/W ratio). After charring, the L/W ratio of rice significantly increased and became the highest, ranging from 1.69 to 1.98. The L/W ratio of wheat gradually changed from 1.82-1.96 before charring to 1.09-1.69 after charring, indicating notable morphological changes during the heating. The length of soybean grains during the charring process maintained stable while its width decreased. The morphological changes of broomcorn millet and foxtail millet were similar in that their width became greater after charring because of the transverse cracking during the charring process. The ratios of the identifiable charred broomcorn millet and foxtail millet in each group gave ranges of 0.75-0.78 and 0.62-0.66, respectively (here, ratio = number of identifiable grains per group/number of samples per group [*n=20*]).

### 3.2 Changes in volume

**Figure 4:**
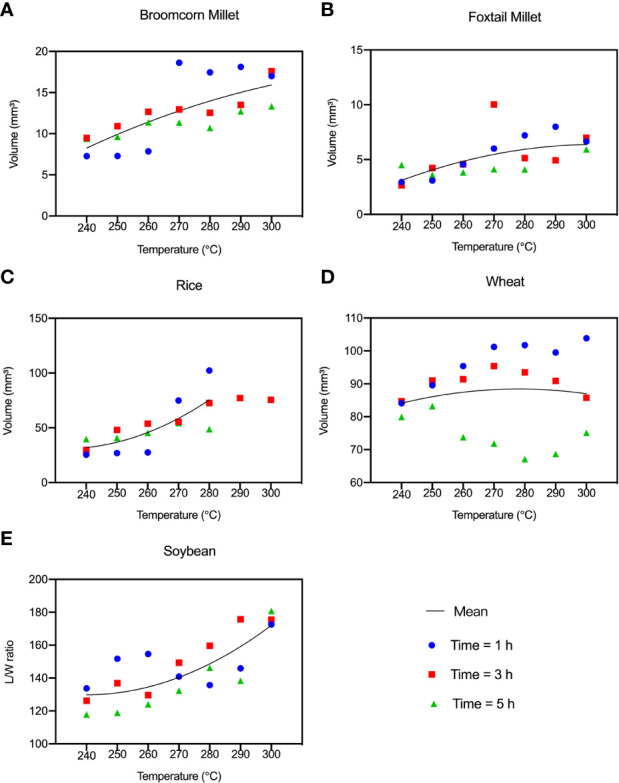
Average volume of the “Five Grains” at different temperatures and times during the heating process. **(A)** Broomcorn millet; **(B)** Foxtail millet; **(C)** Rice; **(D)** Wheat; **(E)** Soybean.

When rice was heated between 290°C and 300°C, it was difficult to verify the geometric characteristics of the grains because the grains were markedly swollen. In identifiable rice grains, the maximum expansion of the volume was between 26.98% and 36.38%. The maximum expansion of the volume of broomcorn millet and foxtail millet was 30.69%-35.1% and 11.97%-15.49%, respectively, while soybeans expanded by 28.79%-31.81%. The wheat grains swelled to the maximum of 19.29% and then shrank to 15.15% of the original volume during the charring process. Wheat has a thick pericarp that hinders the escape of gaseous substances after internal chemical reactions. The volume of the other four grains decreased during the dehydration process and then rapidly expanded during charring (**Figure 4**).

### 3.3 Weight loss

**Figure 5:**
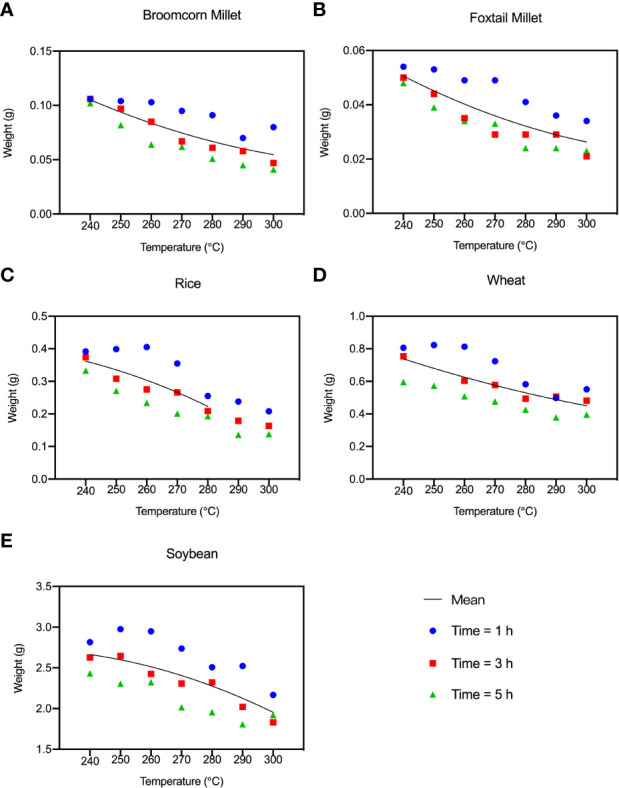
Average weight of the “Five Grains” at different temperatures and times during the heating process. **(A)** Broomcorn millet; **(B)** Foxtail millet; **(C)** Rice; **(D)** Wheat; **(E)** Soybean.

The weight loss of the grains exposed to high temperatures for a short duration is similar to that resulting from prolonged heating at low temperatures. The higher the temperature and the longer the time, the more weight the grain loses. The rate of weight loss of rice, broomcorn millet, foxtail millet and wheat at different heating times varied substantially. However, the weight loss in soybean was highly consistent. The weight loss of identifiable charred rice was 25%-42%. The weight loss of millets and soybean was 45%, and the weight loss of wheat was 45%-50% after charring (**Figure 5**).

### 3.4 SEM observation

**Figure 6:**
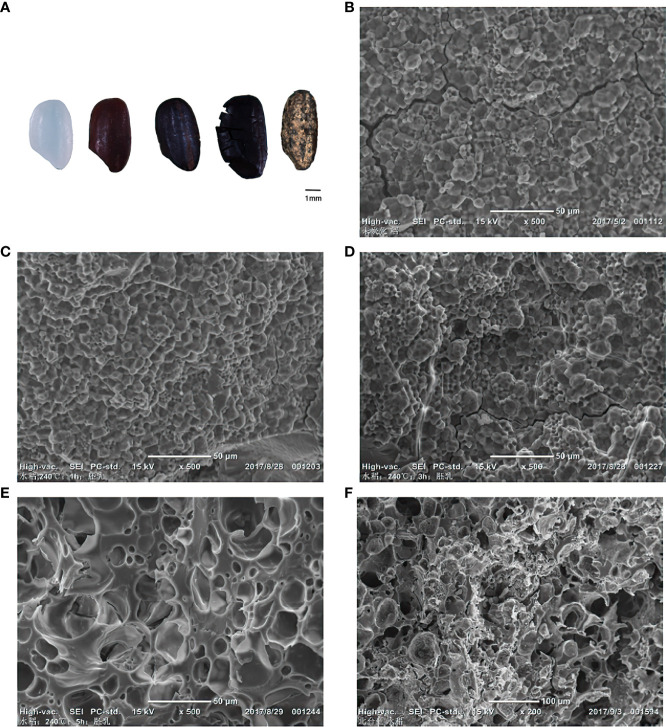
Morphological changes of rice samples under different temperatures and times under SEM. **(A)** Changes in rice morphology during B-F; **(B)** Uncharred rice; **(C)** Rice endosperm after charring at 240°C, 1h; **(D)** Rice endosperm after charring at 240°C, 3h; **(E)** Rice endosperm after charring at 240°C, 5h; **(F)** Rice from archaeological context.

Rice contains water, protein, starch, fat, minerals, and many other nutrients. Starch is the main component and accounts for about 70% of rice grain. The shape of starches is polyhedral and angular under SEM (Shapter et al., 2008). The heating temperature of 240°C is critical for rice charring. At this temperature, the number of holes between starch granules gradually increased. After 5 h of charring, the rice grains burst and gradually lost the identification characteristics. Microscopic observations revealed that the grains became honeycomb-like, and the starch granules were no longer present. When the temperature increased to 270°C or higher, the grains became unrecognizable. Even when heated for only 1 hour at 270°C, the grains exploded rapidly and spurted tar-like substance, and the internal structure confirmed complete charring. The hardness of the grains declined rapidly beyond this temperature point, and they cracked and completely disintegrated at 300°C (**Figure 6**).

**Figure 7:**
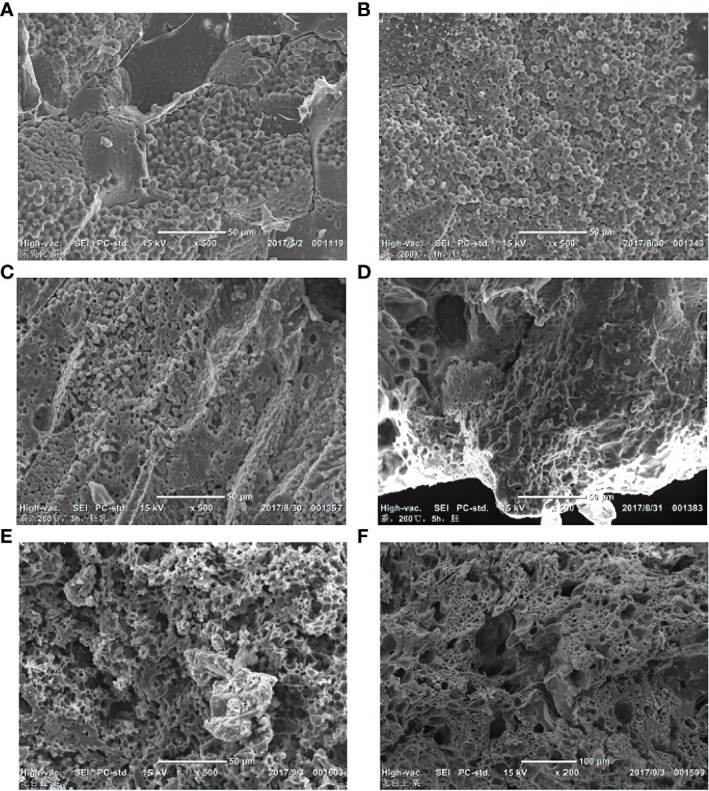
Morphological changes of foxtail and broomcorn millet samples under different temperatures and times under SEM. **(A)** Uncharred broomcorn millet; **(B)** Broomcorn millet endosperm after charring at 260°C, 1h; **(C)** Broomcorn millet endosperm after charring at 260°C, 3h; **(D)** Broomcorn millet embryo after charring at 260°C, 5h; **(E)** Broomcorn millet from archaeological context; **(F)** Foxtail millet from archaeological context.

Broomcorn millet and foxtail millet contain 70% starch, most of which is polyhedral with some other shapes, such as spherical, oval, and irregular. The average size of the starch granules of broomcorn millet is smaller than that of foxtail millet (Yang et al., 2010). In our charring experiments, both the broomcorn and the foxtail millet grains were expanded and became spherical at 260°C. With the increase of the temperature, the grains began to dissolve from the kernel center outwards and gradually became annular. The chemical components were converted to gaseous state. The small holes between starch granules inside the embryo became progressively smaller from its interior towards the outer surface (Yang et al., 2011). After heating for 5 hours, the starch in both millets was gelatinized and charred without any crystal structure. At 280-290°C, the embryos of both millets have similar appearance (**Figure 7**), and no morphological features could be discerned.

**Figure 8:**
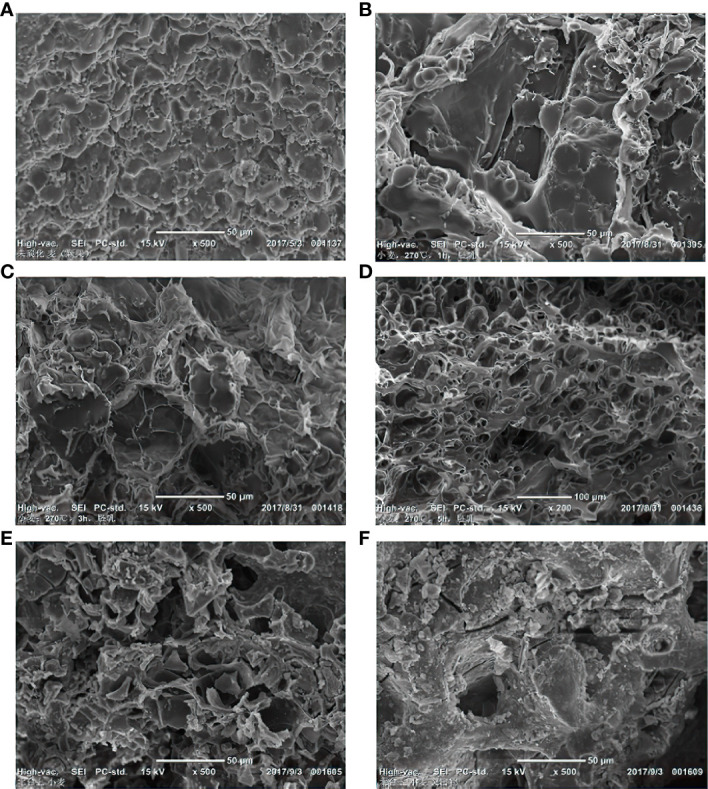
Morphological changes of wheat samples under different temperatures and times under SEM. **(A)** Uncharred wheat; **(B)** Wheat endosperm after charring at 270°C, 1h; **(C)** Wheat endosperm after charring at 270°C, 3h; **(D)** Wheat endosperm after charring at 270°C, 5h; **(E)** Wheat from archaeological context; **(F)** The protrusions on wheat from archaeological context.

Wheat contains about 71% carbohydrates. Larger starch grains of wheat are biconvex/lentoid, while smaller starch grains are spherical. The wheat grains expanded at 270°C, and the inner lentoid and spherical starch granules began to melt (Braadbaart, 2004), while the amount of gelatinized starch gradually increased. The starch grains in the wheat endosperm swelled rapidly at 280°C. Meanwhile, the produced gas built up pressure inside the pericarp, breaking the fragile part of the wheat (mainly the embryo), and releasing volatiles that pushed the partially transformed endosperm outside to form protrusions (Braadbaart et al., 2005). When the temperature continued to increase, the number of protrusions on the wheat grains also increased significantly (**Figure 8**).

**Figure 9:**
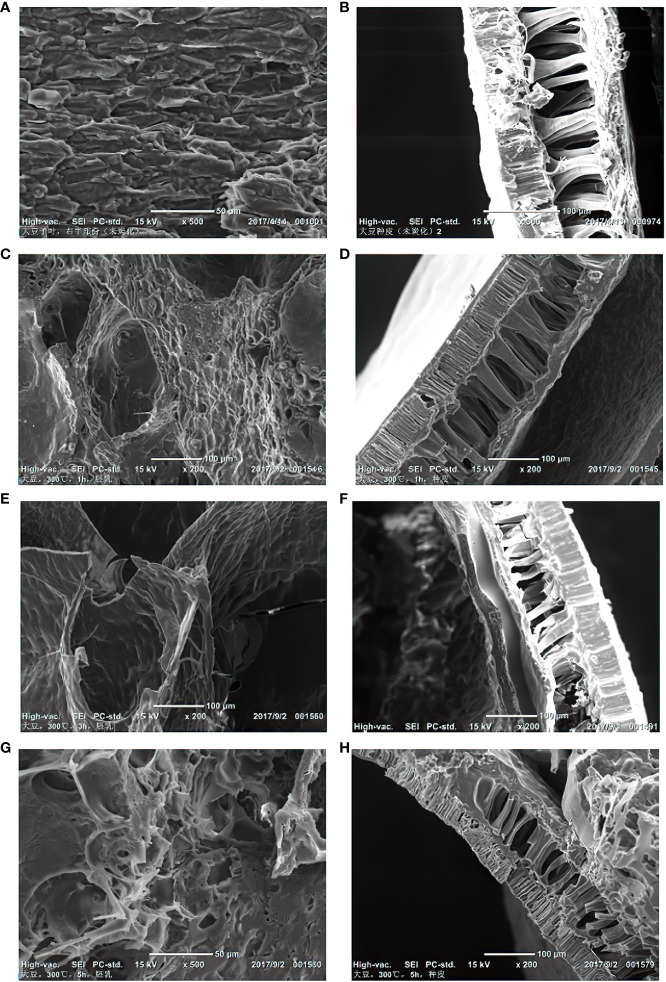
Morphological changes of soybean samples under different temperatures and times under SEM. **(A)** Uncharred soybean cotyledons; **(B)** Uncharred soybean seed coat; **(C)** Soybean endosperm after charring at 300°C, 1h; **(D)** Soybean seed coat after charring at 300°C, 1h; **(E)** Soybean endosperm after charring at 300°C, 3h; **(F)** Soybean seed coat after charring at 300°C, 3h; **(G)** Soybean endosperm after charring at 300°C, 5h; **(H)** Soybean seed coat after charring at 300°C, 5h.

Soybean turned dark brown in color when heated, and the two halves of the cotyledons swelled. The seed coat cracked but stuck to the cotyledons. Heating also changed the protein structure, and the seeds became permeable to oil. Oil can move through the cotyledons to coalesce (Zong et al., 2017). Large oil droplets appeared and holes were created in the charred seeds. As large oil droplets moved through the cotyledons, the surface of the soybean became shiny. After the soybean was heated at 300°C for 5 hours, the interior became a porous structure composed of holes of different sizes, and the seeds were completely charred. The large oil droplets were eliminated from the soybean after charring (**Figure 9**).

### 3.5 Statistical analysis

According to the statistical results (**Table 1**), in general, under the influence of the charring time, temperature, and the interaction between time and temperature, the morphology of “Five Grains” seeds changed significantly (*p* < 0.05). The influence of charring temperature is more significant than that of time, and the interaction between time and temperature is also noticeable.

**Table 1:** Result on Multivariate ANOVA (F value) of the effects of charring time and temperature on various morphological indexes of grains.

Species	Variable	Length variation	Width variation	Thickness variation	L/W ratio variation	Volume variation
Broomcorn Millet (*N*=416)	Time	10.705***	5.161**	7.400***	1.557	24.873***
	Temperature	12.550***	66.645***	34.849***	10.806***	69.102***
	Time*Temperature	4.503***	13.425***	13.689***	2.598**	20.105***
Foxtail Millet (*N*=416)	Time	7.681***	9.342***	10.402***	3.723*	22.626***
	Temperature	6.571***	36.112***	13.381***	11.383***	33.659***
	Time*Temperature	5.171***	12.340***	7.265***	3.584***	17.784***
Rice (*N*=270)	Time	8.952***	1.644	0.344	10.489***	3.824*
	Temperature	47.690***	17.276***	39.346***	6.185***	77.895***
	Time*Temperature	17.362***	8.641***	14.698***	2.496*	30.182***
Wheat (*N*=418)	Time	31.588***	4.810**	3.081*	9.945***	18.210***
	Temperature	30.213***	7.819***	6.483***	41.716***	0.476
	Time*Temperature	1.062	2.788***	1.727	2.628*	1.675
Soybean (*N*=401)	Time	3.065*	6.077**	2.907	13.805***	0.077
	Temperature	31.352***	5.294***	8.223***	33.414***	14.412***
	Time*Temperature	1.663	2.065*	2.363**	3.504***	1.133

*P<0.05, **P<0.01, ***P<0.001.

## 4 Discussion

### 4.1 Factors affecting and affected by the charring process

**Figure 10:**
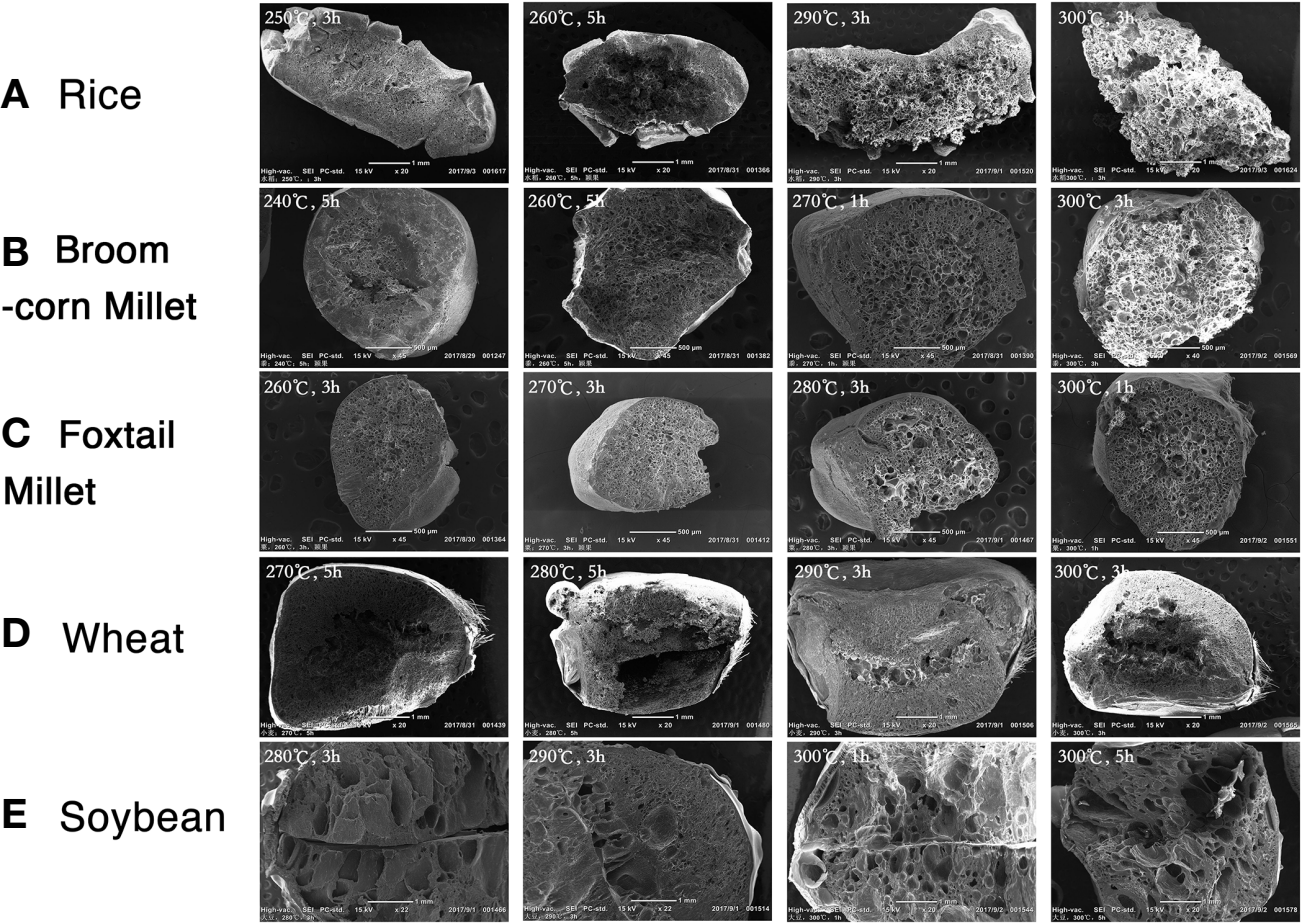
Grain cross-section observed under SEM showed more porosities in the interior of the grains than along the edge. **(A)** Rice; **(B)** Broomcorn millet; **(C)** Foxtail millet; **(D)** Wheat; **(E)** Soybean.

The interior of the grains has more porosities compared with the edge, indicating the interior is the part that becomes charred first during the charring process (**Figure 10**) (Märkle and Rösch, 2008; Yang et al., 2011), despite some studies having shown that charring may start from the exterior and then proceed inward (White et al., 2019). The reason behind this discrepancy may lie in the fact that different species of plants have different chemical composition and different sensitivity to heating, thus exhibiting different responses to charring (Gustafsson, 2000). Different charring conditions may also contribute to these variation. Our discussion revolves mainly around the results of our own experiments, but we acknowledge that there are other possibilities that could cause the same degree of charring, such as a prolonged heating at low-temperature (Charles et al., 2015; White et al., 2019). The overall shape of the grains had undergone some changes during the charring process. The rice grains gained in length, and the wheat grains became rounded. The two kinds of millet grains became larger, and the soybean seeds became flatter.

These results demonstrate that temperature and heating time are essential factors in the charring process. Significant differences were recorded between time, temperature and changes in seed size based on the statistical analysis. Thus, we will discuss the effects of charring from two perspectives: duration and temperature. We use the term ‘shrinkage’ to refer to decrease in the grain dimensions considered (length, width, and thickness).

**Figure 11:**
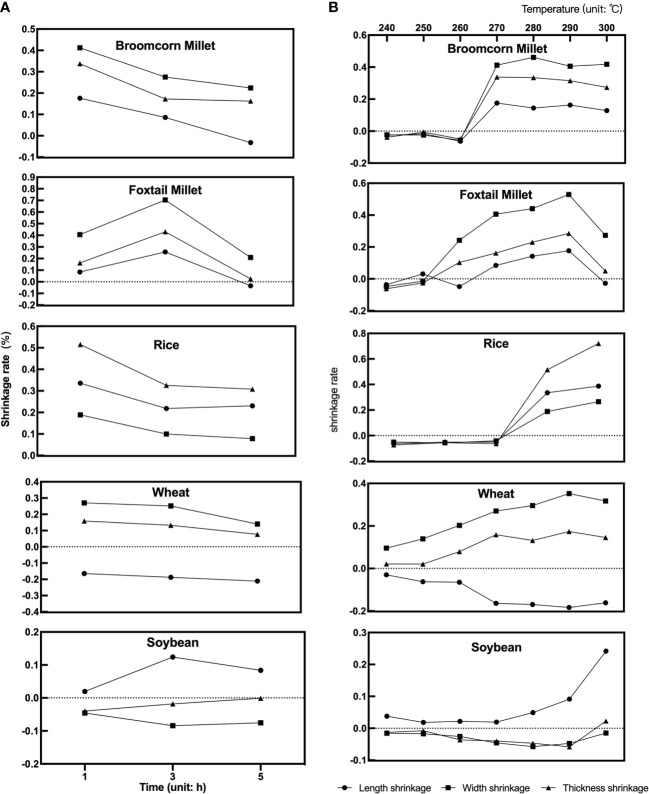
Variation of seed shrinkage under different times and temperatures. **(A)** Change of shrinkage to varying times at 270°C; **(B)** Change of shrinkage to varying temperatures in 1h.

First, to verify the effect of different charring times on the grains, the appearance of the grains was compared when the charring time was set at 1, 3 and 5 h, respectively, while temperature was constant. The grains of “Five Grains” showed different degrees of charring under the same heating time. **Figure 11A** shows the change of the shrinkage rate at 270°C, which is the median value of all charring temperatures. The most considerable variation was observed for foxtail millet, followed by broomcorn millet, rice, and wheat, while the variation of soybean under different charring times was low. The most remarkable change in shrinkage rate was observed from 1 h to 3 h.

Second, to verify how temperatures affected the grains, the shrinkage rate of seed length, width, and thickness was compared at different temperatures after 1 h of charring (**Figure 11B**). It can be seen that the length, width, and thickness of broomcorn millet, foxtail millet, and rice shrank during charring at lower temperatures. Nevertheless, when temperature increased to 260°C, the seeds swelled and then burst as the temperature rose. The length of wheat shrank throughout the charring process and became smaller as the temperature increased, while the width and thickness only slightly increased during the heating process. The shrinkage of length, width and thickness of soybean hardly varied at different temperatures, with a tendency to shrink slightly but then expand when heated to 290-300°C. To sum up, the width of millets underwent the most alteration, and the trend of shrinkage of length, width and thickness tended to be similar under 290°C. The increase in width of millets can also be seen in the F-value which demonstrates that the width of millets was greatly affected by temperature (**Table 1**). Rice and wheat showed the highest variation in thickness and length, respectively, while the length of soybeans did not change too much compared with the other crops.

### 4.2 Weight and density changes caused by the temperature and heating time

Temperature showed a negative correlation with the weight of the grains. Within the experimentally simulated temperature range, the higher the temperature and the longer the heating time, the more weight loss was observed. We hypothesize that grains with high thermotolerance undergo less morphological changes during charring. In this experiment, the change in soybean weight with temperature was the smallest, while that of wheat was the largest.

It is worth noting that the density of all “Five Grains” decreased to varying degrees during the charring process. In general, soybean had the highest density and wheat had the lowest before charring. The density of the “Five Grains” displayed the same trend after charring. Foxtail millet and broomcorn millet had more severe density loss after charring compared with other species, though the differences were not considered significant. Our conclusion is that, the density has little effect on the charring process, but the charring process significantly impacts the seed density.

### 4.3 Temperature range of the charring process

Firstly, a higher degree of charring of the grains was observed when a higher heating temperature was used while the heating duration was kept constant. The charring process of rice was initiated at 240°C for 1 hour. At 270°C or above, it was challenging to preserve the charred rice intact. In archaeological situations, even if the rice grains charred in similar conditions were preserved, it might be hard to recognize them because the high temperature would have destroyed their diagnostic morphological characteristics. The charring process of both foxtail and broomcorn millet started at 260°C for 1 hour or 240°C for 3 to 5 hours. The charred grains were hard to recognize when the temperature reached 280-290°C. Thus, identifying archaeological finds of millets charred above this temperature is a significant challenge. Wheat started to be charred at 270°C for 1 hour or 250°C for 3 hours, and became unrecognizable at 300°C for 5 hours. The protrusions that formed on wheat grains can help determine the temperature of fire, since they appear when the grains are heated over 280°C. Soybean started to show signs of charring at 260°C for 1 hour or 240°C for 3 hours. The seeds became unrecognizable when the temperature was set at 300°C for 5 hours, which is a higher threshold than that for the other four species. This could explain why the charred soybeans survive well in archaeological sites. If the heating temperature is too high, the other types of grains will be burned to ashes, while soybean grains can survive. Therefore, the quantification of plant remains from archaeological sites must be reconsidered. At the very least, our findings show that it could be problematic to discuss the percentages of soybeans relative to the other four grains.

Secondly, the degree of charring of the grains increased with the extension of heating time when the heating temperature was set constant. For example, when the heating temperature was set at 240°C, the wheat grains turned brown and the embryo became lighter in color when heated for 1 hour. After 5 hours of heating, the internal starch grains disappeared, while the exterior cracked and turned dark brown in color. When the heating duration was set at 5 h, rice was the first grain to be charred at 240°C, followed by millets charred at 260°C and wheat at 270°C. Assuming that a wider charring temperature range allows for a higher chance of preservation, soybeans are most likely to be preserved, followed by wheat, rice, and millets. The temperature at which starch granules are modified can be broadly reconstructed through charring experiments followed by SEM-analysis. This can contribute to the archaeological interpretation of starch granules, which should be further investigated in future studies.

We summarized the charring and destruction temperatures in previous and present charring simulation experiments, as shown in **Table 2**. It can be seen that there are relatively more studies on the charring temperature of millets and wheat compared with other species. Due to the differences in the species varieties, temperatures, durations, and oxidation-reduction reactions, there are some differences between these studies. However, in general, the data show similar trends. Previous studies allowed ashing as the end of the experiment, which means the degree of charring differs between these studies and ours. Our focus was on monitoring the changes from the beginning of charring to the loss of identification features, which is more in line with the expectations in archaeobotany. Accordingly, our data can be used to infer the charring temperatures of charred seeds found at archaeological sites.

**Table 2:** Charring and destruction temperature ranges of “Five Grains” in different articles (Temperature control using muffle furnace in the laboratory).

Species	Lowest charring temperature	Lowest destruction temperature	Heating conditions	Reference
Rice (*Oryza sativa*)	240°C in 1h	270/300°C in 1h	Oxidizing	This paper
	180°C in 100min	210°C in 100min	Oxidizing	(Wang et al., 2015b)
	225°C in 45min	275-310°C in 45min	Reducing	(White et al., 2019)
	\	320-330°C	Using thermogravimetric kiln	(Garton, 1979), cited from Castillo, 2019
	250°C	450°C in 2-6h	Oxidizing & reducing	(Chuenwattana, 2010), cited from Castillo, 2019
Foxtail millet (*Setaria italica*)	260°C in 1h	280/290°C in 1h	Oxidizing	This paper
	270-280°C in 0.5-4h	340-390°C in 0.5-4h	Oxidizing	(Wang and Lu, 2020)
	275-315°C in 0.5-4h	325-380°C in 0.5-4h	Reducing	(Wang and Lu, 2020)
	220-235°C	400°C in 4h	Oxidizing	(Märkle and Rösch, 2008)
	\	550-450°C during 1-4h	Reducing	(Märkle and Rösch, 2008)
	215°C	260°C in 4-24h	Restricted access of oxygen	(Dong et al., 2022)
	200-250°C in 0.5h	300°C	Using drying oven	(Yang et al., 2011)
	250°C	300°C	Oxidizing	(Walsh, 2017)
Broomcorn millet (*Panicum miliaceum*)	260°C in 1h	280/290°C in 1h	Oxidizing	This paper
	275-325°C in 0.5-4h	305-325°C in 0.5-4h	Oxidizing	(Wang and Lu, 2020)
	275-305°C in 0.5-4h	305-315°C in 0.5-4h	Reducing	(Wang and Lu, 2020)
	235°C in 1-3h	335°C in 4h	Oxidizing	(Märkle and Rösch, 2008)
	270-245°C in 1-4h	315°C in 4h	Reducing	(Märkle and Rösch, 2008)
	215°C	260°C in 4-24h	Restricted access of oxygen	(Dong et al., 2022)
	200-250°C in 0.5h	250-300°C	Using drying oven	(Yang et al., 2011)
Wheat (*Triticum aestivum*)	270°C in 1h	300°C in 5h	Oxidizing	This paper
	215°C in 40min	315°C in 40min	Oxidizing	(Wang et al., 2015b)
	250°C in 8h	300°C in 1h	Oxidizing	(Su et al., 2019)
	270-310°C in 120min	400-440°C	Anoxic	(Braadbaart et al., 2004)
	310°C	440-600°C	Anoxic	(Braadbaart, 2007)
	250°C in 1.5-2h	350-550°C	Oxidizing & reducing	(Boardman and Jones, 1990)
	215°C	260°C	Reducing	(Nitsch et al., 2015)
	230°C< 12h	250°C >6h	Anoxic	(Berihuete-Azorín et al., 2019)
Soybean (*Glycine max*)	260°C in 1h	Above 300°C in 5h	Oxidizing	This paper
	300°C in 25min	450°C in 25min	Oxidizing	(Zhao and Yang, 2017)

### 4.4 Implications for charred archaeological grains

The effects of charring on seed size, weight and other characteristics have been discussed in previous sections, demonstrating that charring simulation experiments are essential and can help us understand the status of crops. Based on these experiments, we consider some indicators commonly used for crop comparison and attempt to determine when the weight/volume correction should be applied to the quantified remains.

#### 4.4.1 L/W ratio and shrinkage rate

Size is a very important trait in the interpretation of ancient charred seeds. Complemented by other methods, size determination can be used to distinguish cultivated crops from wild species (Fuller and Harvey, 2006), as well as mature grains from immature grains (Fuller et al., 2007; Liu et al., 2007; Song et al., 2013). For example, in studies of agricultural origins, changes in the L/W ratio of rice reflect domestication and provide evidence for the origin of cultivated rice in the lower Yangtze River (Fuller et al., 2007; Liu et al., 2007; Zhao and Gu, 2009). Changes in soybean grains, such as increased grain size and oil content, reflect the influence of human selection (Lee et al., 2011; Wu et al., 2013; Chen et al., 2017). Grain size increases gradually over time, but the change is not uniform (Purugganan and Fuller, 2009). Nevertheless, size is still useful in determining the domestication center and the level of maturity of grains.

**Figure 12:**
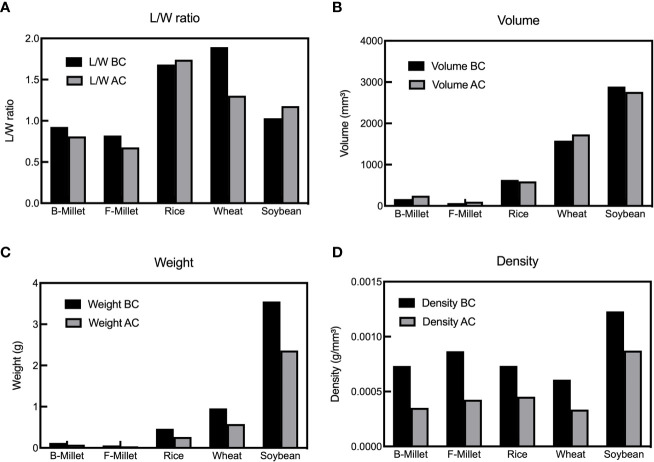
Change of indicators before and after the charring of “Five Grains” (BC=before charring, AC=after charring, B-Millet=Broomcorn Millet, F-Millet=Foxtail Millet). **(A)** The average L/W ratio; **(B)** The average volume; **(C)** The average weight; **(D)** The average density of each group.

**Figure 13:**
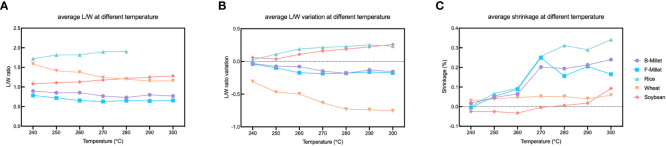
Average L/W and shrinkage ratio at different temperatures. **(A)** The average L/W ratio after charring; **(B)** The average variation of L/W ratio before and after charring; **(C)** The average shrinkage ratio.

The shrinkage rate and L/W ratio are two direct indicators of morphologic changes of seeds which are commonly used to distinguish domesticates from the wild plants (White et al., 2019). **Figure 12** shows that the mean L/W ratios of rice and soybean increased after charring, unlike those of millets and wheat. The use of shrinkage rate as an indicator of the morphological change during charring is still controversial, as White (2019) shows that the average shrinkage of rice is not as large as previously suggested by Fuller et al., 2008 (20%) and states an average width and length reduction of 3.35% and 3.79%, respectively. When charred at 225°C, the L/W ratio of rice remains unchanged. However, our study found that the differences in L/W ratios of rice became more pronounced as the temperature increased. The average shrinkage of length, width and thickness of rice was 18.04%, 7.00%, and 24.78%, respectively, and the overall shrinkage rate of rice is 16%-17%. Our findings are less consistent with previous studies, which may be due to the difference in samples, temperature, and duration. Shrinkage reflects shift in length, width, and thickness, providing a more comprehensive representation of the overall seed morphology compared with the L/W ratio. Fluctuation in the L/W ratio, on the other hand, was relatively indistinct for millets heated at different temperatures, but more noticeable for the other three species (**Figure 13**).

Therefore, in studies relying on the dimensions of rice, wheat, and soybean (e.g., as evidence of domestication), it is critical to keep the impact of charring to a minimum if the L/W ratios are to be explored. One way of doing this is to set a general estimate of the charring temperature of ancient crops or to only compare the seeds from the same archaeological context (assuming that the influence of charring is insignificant within the assemblage from that context). This is because even small variations through the charring process may cause significant discrepancies in the quantified results.

#### 4.4.2 Changes in weight and volume

Since the quantity of macrofossil remains does not directly reflect the level of their presence at a site or the yield of plants, certain corrections to quantifications are needed to more accurately reconstruct the selection of crops in agricultural production. Based on our experiments, we determined under what circumstances the weight or volume measurements of grain assemblages need corrections.

Some existing methods that use modern weight and volume to transfer the absolute number to yield are based on the premise that different crops are preserved to the same degree during and after the charring process (2021; Zhang et al., 2010; Zhou et al., 2016; Sheng et al., 2018). However, as mentioned in Section 4.3, because of the varying survival potential of plants and the fact that the weight of charred seeds varies between different charring and burial conditions, it is difficult to determine the original weight (Yang et al., 2011). Some estimates of wheat and barley yields by weight have taken into account the effects of charring (Ferrio et al., 2004), allowing the reconstruction of original weight and providing a reference for other crops. In addition, statistical methods have been developed by using the weight/volume of 1000 ancient crop grains as the conversion coefficient to estimate the yield, thus reducing the effects of charring (Liu et al., 2019; Jin et al., 2020).

An important application of this approach can be made when determining the ratio of foxtail millet to broomcorn millet. The main identification criterion used to differentiate between these millets is the shape of the embryo; therefore, this element of the grain needs to be well-preserved after charring. Modern broomcorn millet weighs 2.26 times more than modern foxtail millet (Wang and Lu, 2020). The volume of ancient specimens of broomcorn millet grain is 2.74 times higher than that of ancient grains of foxtail millet, based on the samples from a Neolithic site in Shaanxi Province (Liu et al., 2019). In this study, it was found that the average weight and volume of modern millets differ after charring by a factor of 2.08 and 2.42, while before charring these values were 2.05 and 2.38, respectively. Evidently, the effect of charring on the weight and volume of the two millets was insignificant.

In the case of foxtail millet and broomcorn millet, the changes of weight and volume were very similar, so both could be used as a control group in the analysis of the composition of archaeological crop assemblages. The average and total volumes of rice, wheat and soybean grains were relatively consistent before and after charring, while the weight was changed after charring (**Figure 12**). The quantity and weight are susceptible to the charring process and this should be kept in mind when analyzing crop assemblage containing these three species. Their volume is a more appropriate indicator of their representation than the quantity and weight. The volume and weight are more suitable than absolute counts for comparing amounts of grains of individual species within discrete temporal and spatial frameworks. However, due to the significant differences in volume and weight among the “Five Grains”, comparing these parameters between the species has no obvious advantage. In contrast, as a parameter related to weight and volume, the density has a consistent trend among different species (**Figure 12**), and it can thus be used as a measure of representation of different crops in archaeological assemblage. Furthermore, the average density of the “Five Grains” after charring was about 30%-40% lower than before charring, which is then a value that can be used as the density loss rate in conversion.

More experimental studies are needed to explore whether or not the charring process dramatically influences the composition of archaeological crop assemblages. It should also be considered that modern grains have different starch and water contents compared with ancient seeds, so experimental research also has its limitations. In the future, more seeds of different varieties should be investigated.

## 5 Conclusions

We investigated the weight-related, morphological and microscopic changes of several kinds of charred grains, including dehusked rice, broomcorn millet, foxtail millet, wheat, and soybean heated under aerobic conditions at different temperatures ranging from 240°C to 300°C and over periods of 1, 3, and 5 hours. The results show that the charring temperature and duration for rice, foxtail millet, broomcorn millet, wheat, and soybean were 240-300°C for 1 hour, 260-290°C for 1 hour, 260-290°C for 1 hour, 270°C for 1 hour-300°C for 5 hours, and 260°C for 1 hour- 300°C for 5 hours, respectively. Soybean was the type best preserved through the charring process, followed by wheat. In conclusion, the heating temperature and duration are the main factors directly affecting the grains, including their size, volume, and weight.

This paper provides a reference for understanding the influence of charring, and proposes the significance and applicability of L/W, shrinkage, volume, weight, and density when analysing charred grains. L/W ratio and shrinkage help us understand charring-induced changes in the dimensions of the grains, which can be used for disentangling domesticated from wild grain types, determination of the maturation stage of the grains, or distinguishing genera and species of plants. Weight and volume can be used to analyze the causes of variations between the results of macrofossil and microfossil studies, and gain new archaeobotanical insights. Volume can be used as a conversion factor in the quantification of rice, wheat and soybean, and both volume and weight can be used for the quantification of millet.

This study provides a solid foundation for understanding the factors contributing to the charring of the “Five Grains”. Further investigation is still needed to integrate the results of modern simulation experiments and ancient samples along with the application of additional methods, such as the geometric morphometrics. The combined current and future results will enable us to better quantify the human-plant relationship.

## Data availability statement

The original contributions presented in the study are included in the article/**Supplementary Material**. Further inquiries can be directed to the corresponding author.

## Author contributions

YL and YX performed the data analysis and wrote the manuscript. YL performed the analysis with constructive discussions and revised the manuscript. YX organized the experiment. FZ and ZZW performed the statistical analysis. CW and SYY revised the manuscript. XXC designed the research, supervised the experiment and writing, and provided the ancient samples. All authors contributed to the article and approved the submitted version. All authors read and approved the final manuscript.

## Supplementary material

Supplementary Table 1 Average measurement results of the samples.

Supplementary Table 2 Original dataset.

Supplementary Table 3 Detailed statistical results and charring condition of each group.
